# CXCR3 plays a critical role for host protection against Salmonellosis

**DOI:** 10.1038/s41598-017-09150-z

**Published:** 2017-08-31

**Authors:** Belal Chami, Amanda Yeung, Michael Buckland, Hongjun Liu, Genevieve M. Fong, Kun Tao, Shisan Bao

**Affiliations:** 1 Department of Pathology, Tongren Hospital, Shanghai Jiaotong University, Shanghai, China; 2 Discipline of Pathology, School of Medical Sciences and Bosch Institute, The University of Sydney, Sydney, Australia

## Abstract

CXCR3 and its ligands are heavily associated with inflammation and have been implicated in numerous inflammatory diseases. CXCR3 plays an important role in recruiting pro-inflammatory cells, specifically neutrophils, in a model of sterile colitis whereby CXCR3^−/−^ mice showed an attenuated course of colitis with markedly reduced host-tissue damage in the inflamed caecum. The role of CXCR3 during infectious colitis, however, is unclear and therefore in this study, we investigated the role of CXCR3 in the regulation of the immune response during acute and chronic gastrointestinal infection, using a murine model of *Salmonella enterica serovar Enteritidis*. During acute infection with *Salmonella*, we observed significantly increased *Salmonella* loading in the caecum and dissemination to the spleen and liver in CXCR3^−/−^ mice, but not in Wt counterparts. During chronic infection, increased pathological features of inflammation were noted in the spleen and liver, with significantly increased levels of apoptosis in the liver of CXCR3^−/−^ mice, when compared to Wt counterparts. In addition, compromised intestinal IgA levels, CD4^+^ helper T cells and neutrophil recruitment were observed in CXCR3^−/−^ challenged with *Salmonella*, when compared to Wt counterparts. Our data suggests that CXCR3 is a key molecule in host intestinal immunity against Salmonellosis *via* regulating neutrophils chemotaxis.

## Introduction

Chemokines influence the recruitment of leukocytes to sites of inflammation and are implicated in the host protection against numerous infectious agents. Infectious inflammation is usually self-resolving, whereby the reduced infectious stimuli regulates the chemokine response, allowing the appropriate cessation of pro-inflammatory cell influx into inflamed tissue. During sterile inflammation, however, the prolonged stimuli results in the continuous dysregulation of chemokines and subsequent leukocyte recruitment to the inflamed site, resulting in an accumulation of pro-inflammatory leukocytes, thereby exacerbating inflammation and associated host-tissue damage^1, 2^. As a result, chemokine blockage has become an attractive therapeutical approach for many inflammatory diseases including rheumatoid arthritis^3^, multiple sclerosis^4^ and inflammatory bowel disease (IBD)^5^.

CXCR3 is a chemokine receptor implicated in pathogenesis in several chronic inflammatory disorders, e.g. rheumatoid arthritis^6^, multiple sclerosis, and inflammatory bowel disease^7–9^. CXCR3 is constitutively expressed on pro-inflammatory cells, including neutrophils^10^, macrophages^11, 12^, T cells^13^, B cells^7^, and natural killer (NK) cells^14^, while its ligands, CXCL9, CXCL10, and CXCL11 are induced by IFN-γ^15^. Our previous reports demonstrate the pro-inflammatory effects of CXCR3 in DSS-induced colitis – a model of IBD^16^, as well as, in hepatitis B viral chronic hepatitis^17^ or in drug induced liver failure^18^, suggesting an important inflammatory role of CXCR3 in gastrointestinal track. Many investigators have also highlighted the deleterious role of CXCR3 in many inflammatory conditions^2, 5, 19, 20^, implicating its potential as a novel therapeutic target in inflammation-mediated pathologies. However, little is known of its role in host intestinal immunity, particularly against common gram-negative infectious agents.

*Salmonella* species are facultative, gram-negative intra-cellular bacteria harbouring over 2500 serovars. *Salmonella* is the leading cause of bacterial gastroenteritis with morbidity ~1.3 billion cases worldwide^21^. *Salmonella* invades the gastrointestinal epithelium by transiting through M cells overlying Peyer’s patches and drain to the mesenteric lymph nodes (MLN) *via* the lymphatic pathway during development of salmonellosis. Dissemination of salmonella into the bloodstream occurs *via* the thoracic duct where *Salmonella* are mostly removed by macrophages of the reticuloendothelial system (RES) of mainly the spleen, liver, and bone marrow^22, 23^. Immunocompromised individuals with specific defects or blockades of specific cytokines (particularly IL-12/IL-23/IL-17 and TNF) are highly susceptible to developing primary bacteraemic disease and have markedly increased mortality rates, when infected with *Salmonella* ^24^ highlighting the importance of specific inflammatory mediators in preventing Salmonella dissemination.

Herein, we explored the role of CXCR3 in the clearance of live *S. typhimurium* bacteria and in the recruitment of leukocytes during both acute and chronic salmonellosis.

## Results

### Clinical examination

**Figure 1:**
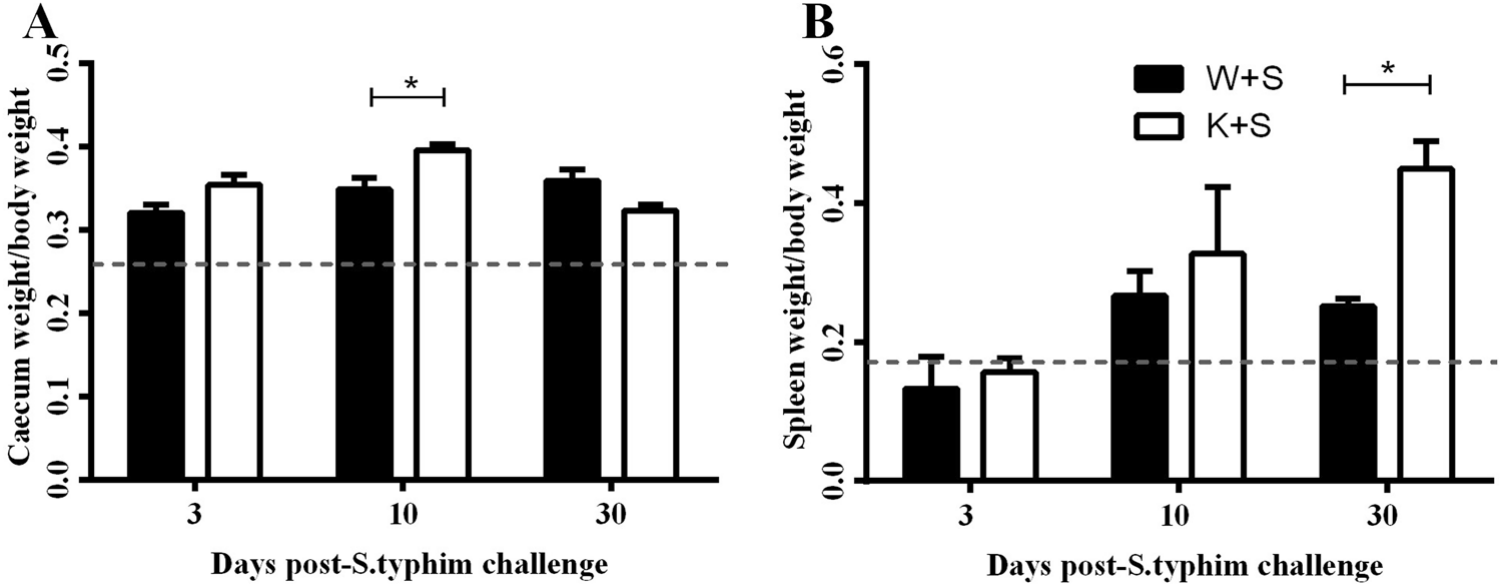
Clinical and post-mortem observations in Wt and KO mice at day 3, 10 and 30 post-*S. typhimurium* challenge. Faecal consistency, hematochesia and rectal bleeding were scored individual and tallied to represent the ratio of (**A**) caecum and (**B**) spleen weight (n = 4) against the respective body weight of mock-treated mice at same time point. Dashed line represents baseline values from combined mock-treated Wt and KO mice. *p < 0.05, and ***p < 0.001. W + S and K + S group values were significantly higher (*p* < 0.05) than baseline at day 3, 10 & 30 in panel A). W + S and K + S group values were significantly higher (*p* < 0.05) than baseline only at day 30 in panel B).

There was no mortality observed in wild-type (Wt) or CXCR3 knockout (KO) mice at days 3, 10, or 30 post-Aro^−/−^ *S. typhimurium* challenge, consistent with our previous observations in this model^25^. At day 3, 10 and 30, *S. typhimurium-*challenged Wt & KO mice showed significantly increased caecum weight to total body weight ratios when compared to their mock-treated counterparts (Fig. 1A), suggesting infectious colitis in the caecum. Interestingly *S. typhimurium*-challenged KO mice revealed significantly heavier caecum at day 10, compared to its *S. typhimurium*-challenged Wt counterpart (*p* < 0.05), suggesting an increased *S. typhimurium* bacterial load in the caecum of *S. typhimurium*-challenged KO. *S. typhimurium* disseminates to the spleen and liver if intestinal mucosal immunity is compromised and/or virulence of the bacteria is strong, where the intestinal infection cannot be contained^22, 23^. Therefore, spleen weight (as a function of body weight) was measurement to assess whether *S. typhimurium* potentially migrated and caused local inflammation to the tissue. Spleen weight increased significantly on day 30 post-*S. typhimurium* challenge in Wt and KO mice, compared to PBS (mock)-challenged group (Fig. 1B). Furthermore, *S. typhimurium*-challenged KO mice had significantly heavier spleens when compared to their Wt counterparts at day 30, suggesting that a more significant degree of bacterial dissemination from the caecum occurred in KO mice. No significant difference was noted between mock-treated Wt and KO mice at any time point.

### Histopathological damage and bacterial load

**Figure 2:**
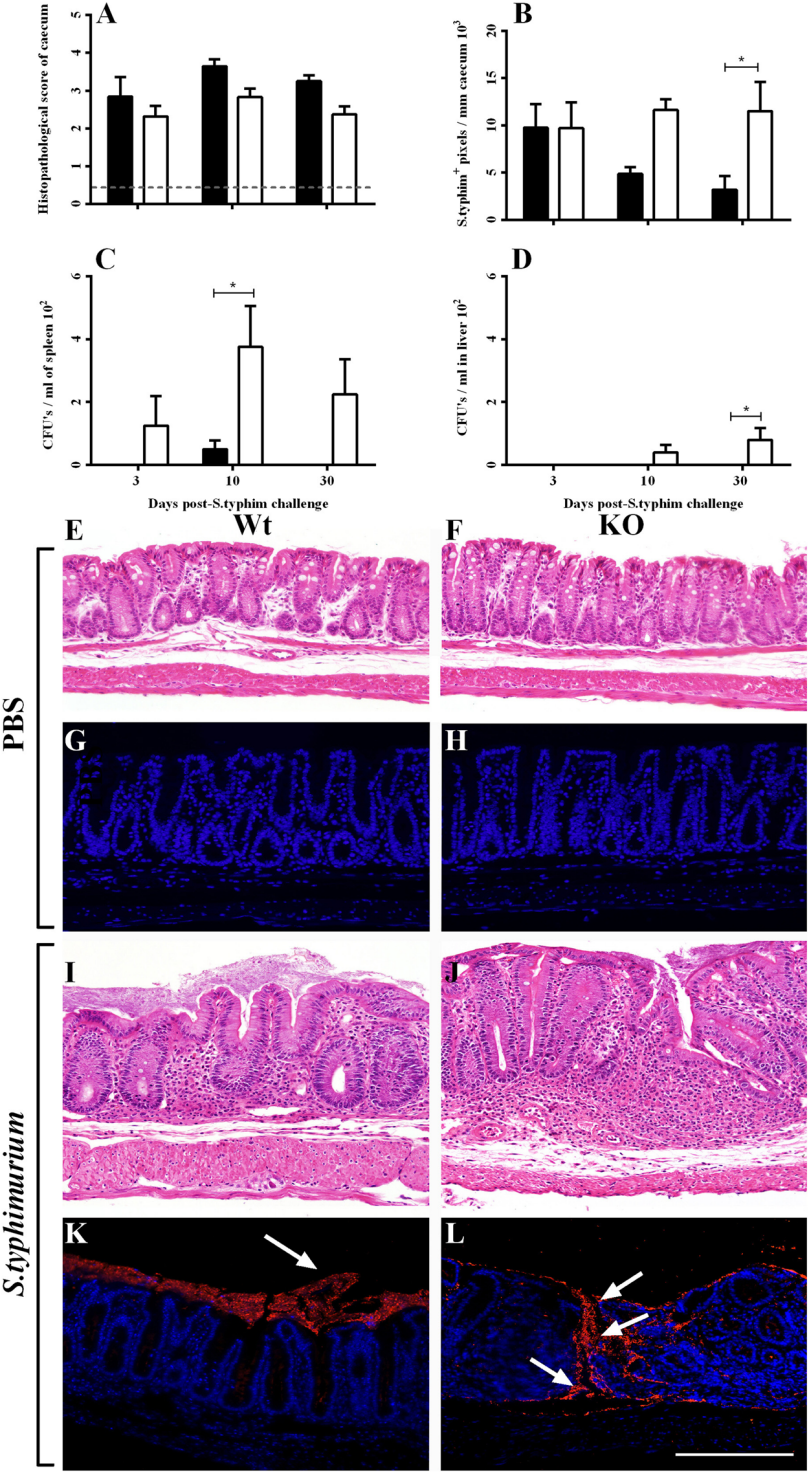
Histopathology and *S. typhimurium* loading in Wt and KO mice at day 3, 10 and 30 post-*S. typhimurium* challenge. (**A**) Histopathological scoring (n = 8) and (**B**) quantification of *S. typhimurium* ^+^ immunofluorescence labelling (n = 4) in the caecum following *S. typhimuirum* challenge. The entire length of the caecum was imaged at 20× magnification and scored or quantified per field of view and later averaged. Enumeration of *S. typhimurium* CFU’s cultured from (**C**) spleen and (**D**) liver homogenates on BBLchrom agar plates (n = 4). Representative H&E and *S. typhimurium* ^+^ immunofluorescence images at day 30 in the caecum of Wt and KO mice treated with (**E–H**) PBS and (**I–L**) *S. typhimurium*, respectively. (**L**) Representative image of *S. typhimurium* breaching the epithelium and lamina propria in *S. typhimuirum* -challenged KO mice. Images were taken at 20× magnification and the scale bar represents 250 µm. Arrows indicate *S. typhimurium* ^+^ labelling. Dashed line represents baseline values from combined mock-treated Wt and KO mice. *p < 0.05 and ***p < 0.001. W + S and K + S group values were significantly higher (*p* < 0.05) than baseline at day 3, 10 & 30 in Fig. 2A.

It was established in our pilot study that salmonellosis did not significantly affect the colon as this region showed little to no pathology following *S. typhimurium* challenge, even after 30 days^26^. However, large areas of inflammatory destruction were detected in the caecum. Histopathological analysis showed that there was significant pathology at day 3, 10, and 30 in both *S. typhimurium*-challenged Wt and KO mice when compared to their respective counterparts (Fig. 2A,I and J), but no signs of pathology in the mock-treated groups (Fig. 2A,E and F). *S. typhimurium*-challenged KO mice showed slightly decreased histopathology at day 3, day 10, and day 30, although this was not significant.

Quantification of *S. typhimurium* in the caecum established the degree of bacterial load in challenged mice. As expected, no detectable salmonella was observed in mock challenged Wt or KO mice (Fig. 2G and H). Furthermore, no differences in *S. typhimurium* ^+^ intensity were noted on day 3 post-*S. typhimurium* challenge in Wt and KO mice (Fig. 2B). However, day 10 and day 30 post-*S. typhimurium* challenge showed increased *S. typhimurium* labelling in the caecum of KO mice, compared to their Wt counterparts (Fig. 2B). This suggests a higher degree of *S. typhimurium* loading in the caecum of KO mice. In addition, most *S. typhimurium* was localised to the caecum lumen in challenged Wt mice, whereas we observed increased transmural *S. typhimurium* labelling in KO mice only, clearly signifying a breakdown in barrier function (Fig. 2K and L). Organs susceptible to bacterial dissemination of *S. typhimurium*, namely, the spleen and liver, were homogenized and cultured for *S. typhimurium*. Cultured spleens homogenates of *S. typhimurium*-challenged KO mice exhibited increased CFUs at day 3, day 10 and day 30 (Fig. 2C), while CFUs in the livers of *S. typhimurium*-challenged KO mice were only found at day 10 and 30 (Fig. 2D). No *S. typhimurium* was detected in the spleen and liver samples of *S. typhimurium*-challenged Wt mice, with the exception of the spleen at day 10 (Fig. 2C).

**Figure 3:**
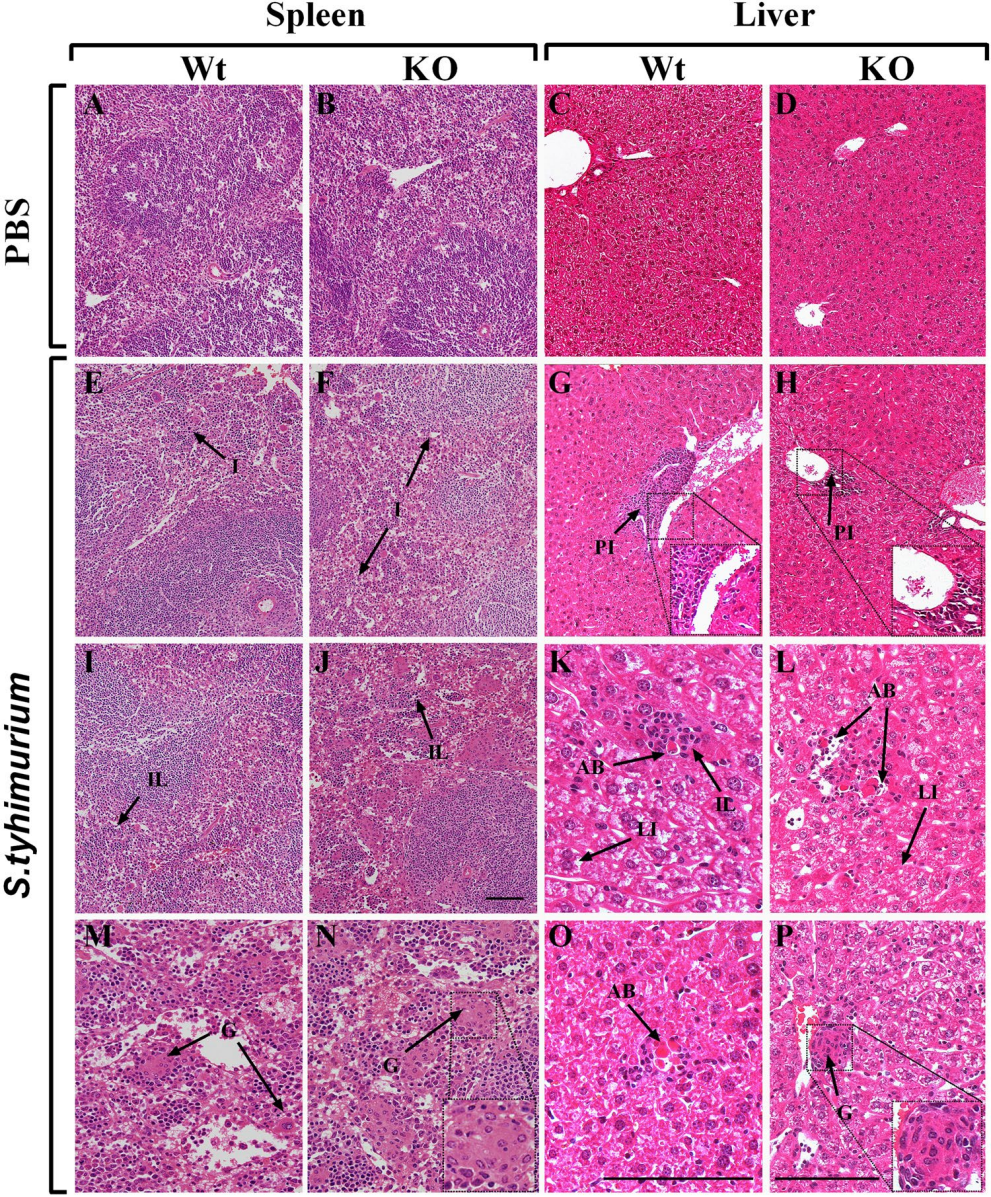
Histopathology of the spleen and liver in mock- and *S. typhimurium*-challenged mice at day 30. (**A**–**D**) H&E images of the spleen and liver in mock-treated Wt and KO mice, respectively. Representative pathological features of inflammation (I) in the spleen of (**E**) Wt and (**F**) KO mice and portal inflammation (PI) in the liver of (**G**) Wt and (**H**) KO mice, challenged with *S. typhimurium*. Lobular inflammation (LI) was also noted in the spleen of *S. typhimurium*-challenged (**I**) Wt and (**J**) KO mice, while acidophil bodies (AB), infiltrating leukocytes (IL) and LI were found in the *S. typhimurium*-challenged liver of (**K**) Wt and (**L**) KO mice. Extensive granulomas (G) were noted in the *S. typhimurium*-challenged spleens of (**M**) Wt and more particularly, (**N**) KO mice. No Gs were found in the liver of *S. typhimuirum*-challenged (**O**) Wt mice, though evidence of Gs were found frequently in (**P**) KO mice. Images A–J were captured at ×20 magnification, L–N & P at ×40 magnification and K & O at ×63 magnification. Scale bar represents 150 µm. All inserts were digitally zoomed 200%.

Next, we assessed the extent of spleen and liver inflammation and determined the histopathology of the respective organs (Fig. 3). Mock-treated mice showed no histological evidence of pathology in the spleen or liver of both groups (Fig. 3A–D). However *S. typhimurium*-challenged Wt and KO mice had regions of inflammation consistent with bacterial dissemination. In the spleen, *S. typhimurium*-challenged KO mice displayed increased regions of inflammation (Fig. 3F), infiltrating lymphocytes (Fig. 3F), and granulomas (Fig. 3N,E,I and M) when compared to Wt counterparts (Fig. 3F,J and N). Histopathological analysis of the liver revealed similar findings to that of the spleen. Granulomas were observed in the livers of *S. typhimurium*-challenged KO mice (P) (Fig. 3G,K and O), which were not observed in the livers of *S. typhimurium*-challenged Wt mice (Fig. 3H,L and P). Although both *S. typhimurium*-challenged Wt and KO mice showed lobular inflammation in the liver (Fig. 3K and L, respectively), the extent of lobular inflammation was observed to be higher in the KO group. In addition, more instances of portal inflammation (Fig. 3H) and leukocyte infiltration were observed in the liver of *S. typhimurium*-challenged KO mice than in livers of Wt counterparts. Acidophil bodies were also present in *S. typhimurium*-challenged Wt and KO mice (Fig. 3K and L, respectively).

**Figure 4:**
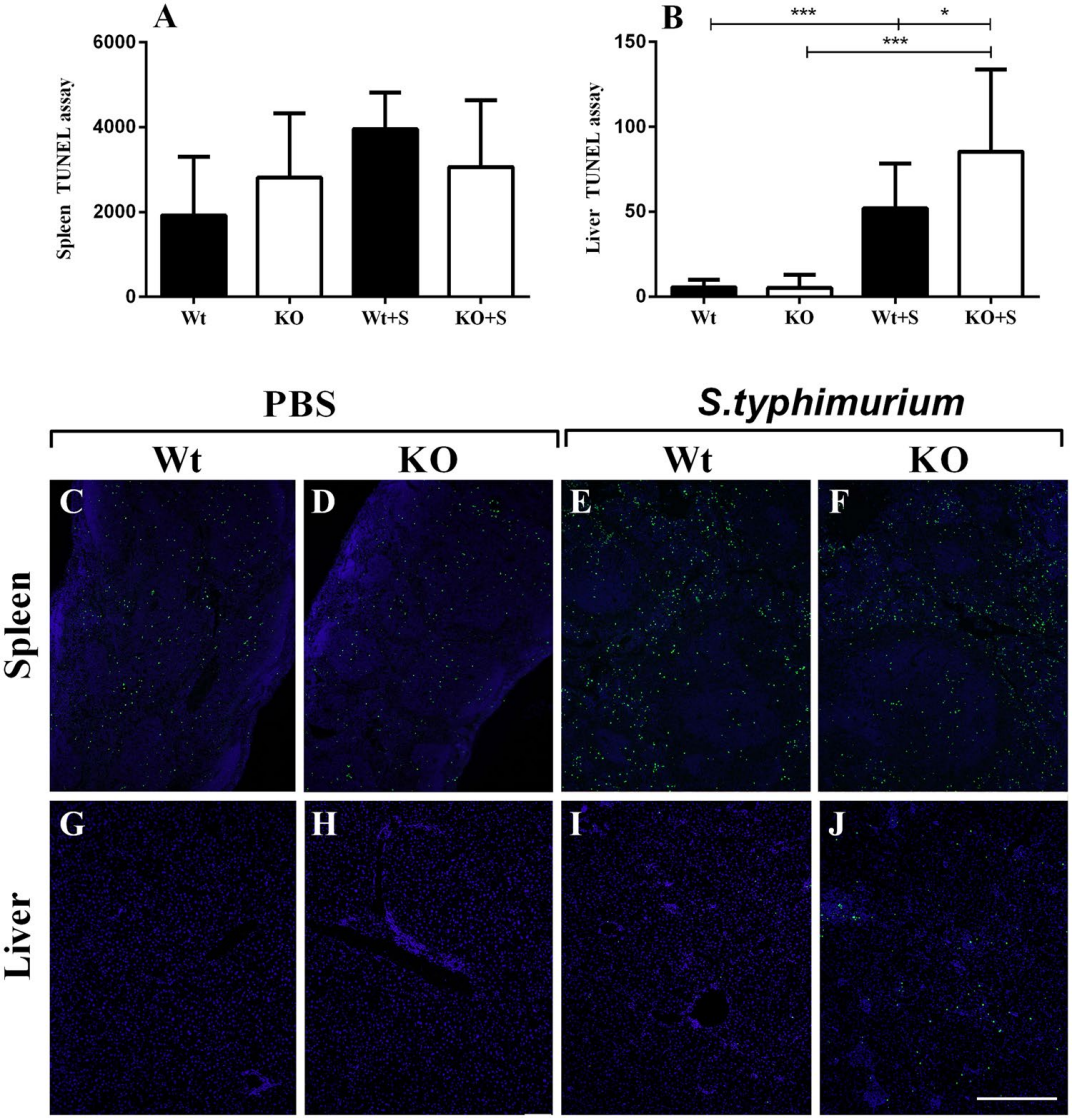
TUNEL assay in the spleen and liver of Wt and KO mice post-*S. typhimurium* challenge. Quantification of positively stained fragmented nuclei in the (**A**) spleen and (**B**) liver of Wt and KO mice at day 30. An average of 10 random microscopic fields at ×20 magnification were captured in the spleen and liver and were each subjected to software-based quantification and represented as mean ± SD. Representative images (×20 magnification) in the spleen in mock-treated (**C**) Wt and (**D**) KO mice and *S. typhimurium*-challenged (**E**) Wt and (**F**) KO mice. Representative images (×20 magnification) in the liver in mock-treated (**G**) Wt and (**H**) KO mice and *S. typhimurium*-challenged (**I**) Wt and (**J**) KO mice. Scale bar represents 250 µm. Data are presented as mean ± SD over 20 microscopic fields (×20 magnification) of each mouse.

Salmonella is a known to hijack macrophages and disseminate to organs in this manner before inducing pyroptosis- A caspase-1 dependent cell death usually induced by intracellular bacteria^27^. We assessed the extent the pyroptosis in the spleen and liver using a TUNEL assay in mice 30 days post-*S. typhimurium*-challenge. No differences in TUNEL staining were noted in the spleens of all groups (Fig. 4A and C–F). However, there was a significant increase in TUNEL staining in the liver of both *S. typhimurium*-challenged groups, when compared to mock-challenged counterparts. (Fig. 4B,G and H). Furthermore, TUNEL staining was significantly increased (*p* < 0.05) in the liver of *S. typhimurium*-challenged KO mice, when compared to the infected Wt mice (Fig. 4B,I and J) – a finding consistent with increased *S. typhimurium* dissemination to the spleen. TUNEL staining in the spleen and liver of mock-challenged Wt and KO mice was consistent and was sparsely observed, especially in the liver (Fig. 4C,D, G and H, respectively).

### Cellular infiltrates

**Figure 5:**
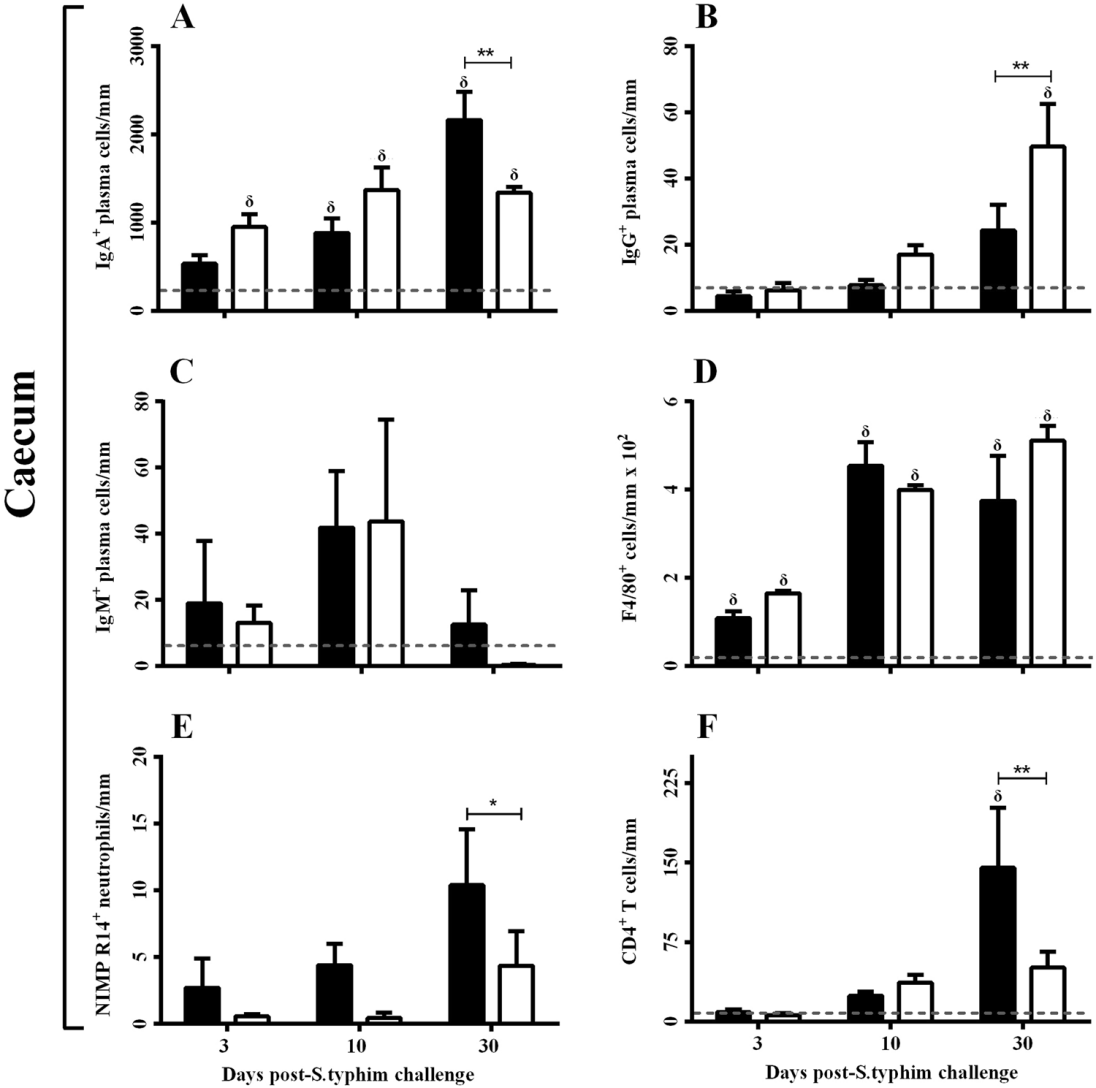
Leukocyte influx to the caecum of *S*. *typhimurium*-challenged Wt and KO mice at day 3, 10 and 30. Software-based enumeration of IHC labelled (**A**) IgA^+^, (**B**) IgG^+^ and (**C**) IgM^+^ plasma cells in the caecum. Macrophage, neutrophil and T cell influx into the caecum was also software-enumerated by IHC labelling of markers (**D**) F4/80^+^, (**E**) NIMP R14^+^ and (**F**) CD4^+^, respectively. Dashed line represents baseline values from combined mock-treated Wt and KO mice. Data are presented as mean ± SD (n = 4) over 20 microscopic fields (×20 magnification) of each mouse. *p < 0.05, **p < 0.01 and ***p < 0.001. W + S and K + S group values were significantly higher (*p* < 0.05) than baseline at day 3, 10 & 30 in Fig. 2A, except for W + S day 3. δ Significantly different (*p* < 0.05) than baseline levels.

Reduced migration of CXCR3^+^ B cells can exacerbate salmonellosis, as indicated by a report stating that IgA antibodies against *S. typhimurium* O-antigen (a component of lipopolysaccharide) was highly effective in preventing Salmonella infection^28, 29^. In light of this, plasma cells of several immunoglobulins were quantified using immunohistochemistry. IgA^+^ plasma cells in the caecum significantly increased following *S. typhimurium*-challenge in both Wt and KO mice relative to mock-treated controls, with the exception of Wt mice at day 3 post-infection (Fig. 5A). However, IgA^+^ plasma cells were significantly reduced in *S. typhimurium*-challenged KO mice at day 30 compared to the Wt mice. Interestingly, IgG^+^ plasma cells in the caecum showed a significant increase at day 30 in *S. typhimurium-*challenged KO mice (Fig. 5B). IgM^+^ plasma cells in the caecum appeared to peak at day 10 in both the *S. typhimurium-*challenged groups and, as expected, decreased towards baseline levels by day 30 as the immune response matured (Fig. 5C).

Macrophages, also express CXCR3, play an important role in controlling salmonellosis. Quantification of F4/80^+^ macrophages in the caecum showed no significant differences in *S. typhimurium*-challenged Wt and KO mice, despite the levels of F4/80^+^ macrophages being significantly increased in both groups when compared to their mock-treated counterparts (Fig. 5D), alluding to compensatory mechanisms for macrophage chemotaxis outside the CXCR3-axis. CXCR3 is expressed abundantly on neutrophils and has been previously shown to influence recruitment of neutrophils during colitis^16^. It has also been previously shown that increased intestinal bacterial loading of *S. typhimurium* is associated with compromised neutrophil response^30^, whereby neutrophil recruitment is crucial in limiting *S. typhimurium* infection to the intestine and preventing dissemination^31^. The influx of NIMP R14^+^ neutrophils was reduced in the caecum at day 3, 10, and significantly on day 30 post-*S. typhimurium* challenge, compared to the Wt counterparts (Fig. 5E), suggesting that the CXCR3-axis is crucial for chemotaxis of neutrophils during infectious inflammation. CD4^+^ T cell cells were also significantly mitigated at day 30 in *S. typhimurium*-challenged KO mice relative to Wt counterparts (Fig. 5F).

## Discussion

Dissemination of *S. typhimurium* from the gastrointestinal tract is a potentially life-threatening condition, mediated by neutrophil breakdown barrier^32^ and modified of macrophage motility^33^ It has been reported that the C57BL/6 strain (NRAMP1^−/−^) is susceptible to Salmonella challenge, due to dysfunction of Nrampl^34^. This is in line with our current finding, showing that host response to Salmonella was over 30 days. Furthermore, the mice challenged with the avirulent strain of Salmonella, BRD509, had zero mortality at day 30 post-challenge. Here, we demonstrated CXCR3 is important in gut mucosal immunity to *S. typhimurium* challenge. The absence of CXCR3 promotes bacterial propagation and dissemination, significantly compromising host resistance to infection. This was supported by worse histopathology in liver and spleen, with increased apoptosis. Previously, we have demonstrated that lack of CXCR3 attenuates DSS-induced colitis, possibly owing to limited chemotaxis of proinflammatory cells such as neutrophils and macrophages^16^. However, in the context of intracellular bacteria-induced colitis, CXCR3 seems to be crucial in mounting an efficient immune response to limit and clear salmonellosis, which may be due to host response to different pathogenic challenges.

The AroA/D mutant strain used in the manuscript was obtained from Prof Strugnell^35^, who has described in details how the BRD509 sub-strain was constructed and performed a Southern blot to confirm that BRD509 is deficient in AroA and AroD.

The Wt mice challenged with BRD509 were largely able to contain the infection to within the caecum, which is characteristic of the BRD509 course of Salmonellosis in mice^36^. Moreover, Dunstan and colleagues did not report any mortalities in mice infected with BRD509, even following 38 days post infection and 10 days post boost infection. However, others^26, 37^ have demonstrated that Wt mice infected with the non-attenuated form of Salmonella (SL1344) rapidly succumb to Salmonella infection and do not survive to day 10 post-*S. typhimurium* challenge. We reported zero mortality at day 30 in Wt mice challenged with BRD509, suggesting that the sub-strain did not revert to the virulent SL1344 form.

There was compromised host mucosal immunity in CXCR3 KO mice to *S. typhimurium* challenge in our current study, showing delayed bacterial clearance in caecum, as well as in the liver and spleen. The protective role of CXCR3 during salmonellosis may be attributed to its neutrophil chemotactic function during infection with gram-negative intracellular bacteria. Recently, *S. typhimurium*-induced intestinal injury^38^ and dissemination^32^ is associated with CXCR3 expressing neutrophils and mitigated neutrophil chemotaxis, respectively. In addition, *S.typhimuirum* dissemination to the spleen occurred as early as day 3 in CXCR3 KO mice and was associated with mitigated neutrophil chemotaxis. Neutrophils are a chief source of myeloperoxidase (MPO) in the gut mucosa, which produces hypochlorous acid or other hypohalites from hydrogen peroxide, killing intra-and extracellular bacteria. However, a recent study in MPO KO mice illustrates only a slight increase in *S. typhimurium* survival in neutrophils^39^, suggesting that either MPO may be not a crucial for *S. typhimurium* killing, or that neutrophils can compensate with other oxidative species, such as H_2_O_2_ via reactive oxygen burst to mediate *S. typhimurium* clearance. Interestingly, Cheminary, 2004 purported that rapid neutrophil chemotaxis effectively prohibited *Salmonella* dissemination from the gastrointestinal source, while a mitigated neutrophil response resulted in invasion and destruction of M cells in the PP and subsequent dissemination to various organs^40^. CXCR3-mediated neutrophil migration to the mucosa seems particularly important during the early stages of infection for localising and preventing *S. typhimurium* dissemination, however its significance during chronic Salmonellosis remains unclear.

Intestinal mucosal humoral immunity is critical in host defence against pathogenic invasion. Unlike other immunoglobins, pathogens neutralised with secretory IgA are not subjected to a coordinated immune response, and therefore the likely function of IgA is immune exclusion by preventing the adherence of pathogens to the mucosal surface. Secretory IgA also has a direct effect on the virulence of *S. typhimurium*, binding to the O-antigen component of membrane LPS, destabilising and decreasing flagellum-based motility^41^. Our data showed a significant decrease in caecum IgA^+^ plasma cells in *S. typhimurium*-challenged KO mice at day 30, compared to Wt counterparts. This is consistent histopathology and bacteriology observations in *S. typhimurium*-challenged KO mice, supporting a critical role of IgA in eliminating Salmonella^25^. The compromised mucosal IgA probably is compensated by over production of IgG in the CXCR3 KO mice, despite this, it is not efficient in elimination of *S. typhimurium*. No significant differences of intestinal mucosal IgM^+^ plasma cells between CXCR3 KO and Wt mice were noted, suggesting CXCR3 might not play an important role in regulating host immunity at Ig class switching.

Notably, the possibility exists that the mice used in these experiments may have been exposed to a previous Salmonella infection, despite being held in a specific-pathogen-free (SPF) facility. For example, the mother of the experimental mice may have transferred some level of IgA-mediated immunity to their offspring. Nevertheless, both Wt and KO mice were bred and held in the same SPF facility and any previous exposure to any strain of Salmonella would have been equal.

There is differential regulation of neutrophil and macrophage chemotaxis during *Salmonellosis* in CXCR3 KO mice, despite CXCR3 being expressed on both neutrophils and macrophages^10, 12^. Macrophages may use other compensatory mechanism for activation and/or recruitment in the absence of CXCR3 receptor. Our explanation for the current finding is that substantially reduced IgA but without compromised infiltrating number of macrophage in CXCR3 KO mice, invites the speculation that macrophages alone may not be able to effectively clear *Salmonella*. This is supported by our previous publication, illustrating that bactericidal activity of macrophage is compromised in the absence of IFN-γ^25^.

## Conclusion

Our results suggest that CXCR3 confers early protection against *S. typhimurium* infection which is crucial in preventing bacterial dissemination to the spleen and liver of mice. Both the early recruitment of CXCR3-expressing neutrophils and CXCR3-mediated IFN-γ secretion are instrumental in an effective immune response against gram-negative *S. typhimurium*. Careful consideration of CXCR3’s role in host-immunity is warranted to adequately make a benefit versus potential risk assessment before considering targeting CXCR3 or the ligands in the clinical setting.

## Materials and Methods

### Mice

All experiments were approved and carried out in accordance to The University of Sydney Animal Ethics Committee (AEC) (K20/4-2011/2/5369). The strain for both of Wt and KO mice used in this study was C57BL/6 stain (NRAMP1^−/−^). Wt and Ko mice were bred in the Animal facility, The University of Sydney, and housed in environmentally enriched cages with *ad libitum*. The genetic background was routinely checked in the laboratory animal facility at the time when we obtained the mice^42^. Clinical scores evaluation were performed daily to monitor disease progression and animal wellbeing. Four age- and sex-matched mice were allocated to each group and experiments performed 3 times.

### Inoculation with *S. typhimurium* and Salmonellosis

The recent development of the streptomycin pre-treated murine model of *Salmonella enterica* serovar typhimurium (*S. typhimurium*) has enabled investigation into the molecular mechanisms of invasion, evasion, intracellular survival, and persistence of *S. typhimurium* in host tissue^43^. The attenuated sub-strain, BRD509 (aroA/D deleted mutant) of *S. typhimurium* (strain SL1344) was kindly provided by Professor Richard Strugnell (Department of Microbiology, University of Melbourne, Australia)^35^. Salmonella was positively identified via growth on selective and differential BBL™ CHROMagar™ Salmonella medium (BD, Australia), which specifically colours Salmonella colonies mauve. The strain of Salmonella used was the streptomycin-resistant BRD509 AroA/D mutant, a substrain of SL1344 and grown in streptomycin supplemented lysogeny broth (w/v, 100 µg/mL). C57BL/6 mice (NRAMP1^−/−^) were anaesthetized with inhaled isoflurane (Lyppard, Australia) and orally gavaged with 200 μL 0.75% sodium bicarbonate solution to ensure neutralisation of stomach acid^34^. Both Wt and KO mice were on a C57BL/6 background^42^ and were gavaged with streptomycin (120 µg/1600 uL w/v)^43^ 30 min prior to *S. typhimurium* inoculation. Mice in the *S. typhimurium*-challenged groups were gavaged with 2 × 10^9^/150 µL CFU *S. typhimurium*. Wt and KO mock challenged mice were gavaged with 150 μL of sterile PBS.

### Microbiological Methods

To determine bacterial load within susceptible tissues, spleen and liver from both Wt and KO groups were collected aseptically and pushed through a 0.75-μm cell strainer (BD Biosciences, USA) into 2 mL of sterile PBS. The resulting homogenate was collected, serially diluted (10-fold) in sterile PBS and plated onto LB agar. LB agar plates were incubated overnight in a 37 °C incubator and bacterial growths were counted the following day. Bacterial counts were expressed as CFU per mL of homogenate.

### Caecum Homogenate

The caecum was excised from the ascending colon and small intestine immediately following culling of mice on day 3, 10 and 30 post *S. typhimurium*- or mock-challenge Wt and KO mice. Caecums were examined grossly, weighed and sectioned coronally into two equal sized portions which were either snap frozen in liquid nitrogen or fixed in 70% ethanol for histological analysis. Snap-frozen caecums were later homogenized.

### Histological Examination

The caecum was perfused with cold PBS, weighted, sectioned coronally and stained with haematoxylin and eosin for assessment of histopathological changes. The caecum was significantly affected following *S. typhimurium* challenge at days 3, 10 and 30. Twenty fields of view (×20 magnification) were randomly captured in the caecum under an Olympus BX40 microscope. The images were scored in a blinded fashion and the values averaged. The histopathological scoring method was adopted from previously described methods^44^. Each histological feature, including crypts, epithelia, goblet cells, cellular infiltration and oedema was individually scored to reflect either normal morphology (0–1), mild (2–4), moderate (5–7) or severe (8–10) colitis and tallied.

### Immunohistochemistry

Briefly, specimens were dewaxed, rehydrated, endogenous peroxided activity blocked and incubated with either polyclonal anti-IgA, -IgG, -IgM (1:200 v/v, Merck Millipore), -F4/80 (1:250 v/v, BM8, Life Technologies, Australia), -NIMP R14 (Abcam, England), or anti-CD4 (1:200 v/v, GK1.5, Biolegend, USA) for 1 h at room temperature. Labelling was visualised by peroxidase activity of HRP-conjugated secondary antibody with DAB substrate to form a brown precipitate. Sections were imaged and quantified as previously described^45^.

### Apoptotic Assay

Apoptotic cells in the spleen and liver was assessed using the DeadEnd™ Fluorometric TUNEL System (Promega, USA) in both *S. typhimurium* and mock challenged Wt and KO. Fragmented nuclear DNA of apoptotic cells is measured by catalytically incorporating fluorescein-12-dUTP at 3′-OH DNA ends using the enzyme Terminal Deoxynucleotidyl Transferase (TdT), which forms a polymeric tail using the principle of the TUNEL (TdT-mediated dUTP Nick-End Labeling) assay. The fluorescein-12-dUTP-labeled DNA is then visualised directly by fluorescence microscopy (Olympus BX40 microscope, attached to a digital camera (DP70, Olympus)). Following dewaxing and rehydrating, specimens were permeabilised in 0.2% Triton X-100 in PBS for 5 min. Slides were then rinsed and tissue specimens incubated with 100 μL of Equilibration Buffer 10 min at room temperature (RT). Next, the Equilibration Buffer was removed and 50 µl of TdT reaction mix added to tissue specimens and allowed to incubate for 60 min at 37 °C. Excess TdT reaction mix was removed and slides were immersed in 2X SSC solution for 15 min. Slides were then washed, counterstained in DAPI and coverslipped.

### Photography

An Olympus BX40 microscope attached to a digital camera (DP70, Olympus) was used to photograph slides at 20× and 40× magnification. Images were recorded using Olympus DP controller.

### Statistics

All data are presented as mean ± SD. Statistical analysis was performed using a two-way ANOVA, one-way ANOVA or *t*-test as appropriate. *P*-values less than 0.05 were regarded as statistically significant. Asterisks denote various *p* values as follows: *p < 0.05, **p < 0.01 and ***p < 0.001.
